# Electrochemical Determination of Antioxidant Capacity of Traditional Homemade Fruit Vinegars Produced with Double Spontaneous Fermentation

**DOI:** 10.3390/microorganisms9091946

**Published:** 2021-09-13

**Authors:** Maja Chochevska, Elizabeta Jančovska Seniceva, Sanja Kostadinović Veličkovska, Galaba Naumova-Leţia, Valentin Mirčeski, João Miguel F. Rocha, Tuba Esatbeyoglu

**Affiliations:** 1 Faculty of Medical Science, University “Goce Delčev”, Krste Misirkov bb, 2000 Štip, North Macedonia; maja.cocevska@ugd.edu.mk; 2 Food and Veterinary Agency, III Makedonska Brigada No. 20, 1000 Skopje, North Macedonia; elizabeta_jancovska@yahoo.com; 3 Faculty of Agriculture, University “Goce Delčev”, Krste Misirkov bb, 2000 Štip, North Macedonia; sanja.kostadinovik@ugd.edu.mk; 4 Faculty of Chemistry and Chemical Engineering, Babeş-Bolyai University, 11 Arany Janos Str., 400028 Cluj-Napoca, Romania; gabi_naumova@hotmail.com; 5 Institute of Chemistry, Ss. Cyril and Methodius University, Arhimedova 5, 1000 Skopje, North Macedonia; valentinmirceski@yahoo.com; 6 Department of Inorganic and Analytical Chemistry, University of Lodz, Tamka 12, 91-403 Lodz, Poland; 7 LEPABE—Laboratory for Process Engineering, Environment, Biotechnology and Energy, Department of Chemical Engineering (DEQ), Faculty of Engineering, University of Porto (FEUP), Rua Roberto Frias, s/n, P-4200-465 Porto, Portugal; jmfrocha@fe.up.pt; 8 Institute of Food Science and Human Nutrition, Gottfried Wilhelm Leibniz University Hannover, Am Kleinen Felde 30, 30167 Hannover, Germany

**Keywords:** antioxidant activity, fruit vinegars, cyclic voltammetry, Trolox equivalent antioxidant capacity (TEAC) assay

## Abstract

In the current study, the antioxidant activity of traditional homemade fruit vinegars (HMV) was estimated by measuring the rate of homogeneous redox reaction with 2,2′-azino-bis-3-ethylbenzothiazoline-6-sulfonic acid radical cation (ABTS^•+^) using cyclic voltammetry. The antioxidant capacity of six HMV produced using traditional methods and the physicochemical characterization were measured in different vinegar production steps throughout a double spontaneous fermentation process, i.e., without any addition of yeasts or acetic acid bacteria. Their antioxidant capacity was compared with seven fruit commercial vinegars (ComV). Furthermore, the antioxidant capacity was independently measured with the TEAC (Trolox equivalent antioxidant capacity) assay, aiming at correlating with the electrochemical experimental data. Obtained results from both methods, the electrochemical and TEAC assays, interestingly indicated that all HMV have at least 10 times higher antioxidant activity than ComV. Furthermore, the large range of values for antioxidant capacity in samples of commercial vinegars from apples attested the importance of the raw material quality and technological procedures. The positive correlation between total phenolic content and antioxidant capacity measured by the two type of assays indicated that rose hip homemade vinegar (HMV5) has the highest antioxidant capacity. In contrast, the lowest levels of phenolic compounds and antioxidant capacity were found in apple and persimmon homemade vinegars (HMV1 and HMV6, respectively) which indicated that the type of fruit is crucial towards the production of high-quality vinegars. In this way, the use of traditional processes for the production of fruit vinegars proved to be very promising in terms of producing differentiated vinegars and, concomitantly, reaching high levels of health-promoting antioxidant capacities.

## 1. Introduction

Vinegar is a 5–20% (*v*/*v*) acetic acid (or ethanoic acid, CH₃COOH) aqueous solution, containing a large number of organic substances such as carbohydrates, alcohols, organic acids, volatile compounds and amino acids. Fruit vinegars contain high concentrations of polyphenolic compounds and their final quality depends mainly on the raw material used as a substrate, acidification process, fermentation and ageing procedures employed during manufacturing [1,2,3,4,5]. In addition to the raw materials, aromas and flavors found in different vinegars are mainly a consequence of the traditional methods undertaken for its production—based on a slow acetification and ageing in oak barrels [6,7,8]. One of the most traditional procedures used for vinegar production is the conventional Orleans method, performed commonly at home by using a larger amount of fruits, resulting in high-quality vinegars [9,10,11,12].

Numerous studies have been focused on the antioxidant capacity of traditional and commercial vinegars—such as the most globally consumed apple cider, fruit and balsamic, wine and grape vinegars [13,14,15,16,17,18,19]. Cyclic voltammetry (CV), based on a redox mechanism, is a highly suitable technique for the quantification of antioxidant capacity of a variety of plant extracts and products, wines, fruits, vegetables and herbs, etc. [20,21,22,23,24,25].

This study involved two main aims. The first aim was the comparison of antioxidant capacity of commercial and homemade vinegars from apples, raspberries, blackberries, blueberries, rose hips and persimmons native from the region of North Macedonia, and, simultaneously, the comparison with seven commercial vinegars (ComV). The second aim was to show the effectiveness and usefulness of a new approach for the determination of antioxidant capacity of vinegars by cyclic voltammetry—a fast and non-destructive technique, which enables the obtention of reliable results in comparison to other time consuming antioxidant assays.

A slow traditional process of spontaneous fermentation was employed, i.e., a fermentation process is undertaken by the means of the natural microbiota present in the fruits and without any addition of yeasts or bacteria. Such an artisanal method to produce vinegar was employed to better ascertain the potential of autochthonous microbiota to produce differentiated and high-quality vinegars in comparison to the existing commercial ones, while contributing to the general microbial diversity and protection of microbial heritage. Different environmental factors may substantially lead to variations in the microbial community composition. Several studies on microbial composition in traditional homemade vinegars have been reported, and results suggest that *Saccharomyces* spp., lactic acid bacteria (LAB) and acetic acid bacteria (AAB) are dominant functional microorganisms. According to the findings of the research group of Yun (2019) [26], not only naturally selected microbial species gradually grow up and reproduce in the fermentation process, but also promoters of vinegar fermentation do. The Liangzhou fumigated vinegar fermentation is an example of a continuous process with spontaneous microbial growth that affects the dynamics of the microbial communities present therein. Continuous changes of microbiological environment conditions in the substrate affect the diversity of microbiota [26]. It is expected that the exploitation of the manufacture of vinegars by the use of ancestral methodologies, characterized by spontaneous and slow fermentations, may yield differentiated products with greater commercial added-value and nutritional value.

## 2. Materials and Methods

### 2.1. Chemicals and Reagents

ABTS (2,2′-azino-bis-3-ethylbenzothiazoline-6-sulfonic acid), acetic acid, potassium chloride, ammonium persulfate, α-tocopherol, Trolox (6-hydroxy-2,5,7,8-tetramethylchroman-2-carboxylic acid), ascorbic acid, and gallic acid were obtained from Sigma-Aldrich (Seelze, Germany), Fluka Chemie (Buchs, Switzerland) and Merck (Darmstadt, Germany). All chemicals were analytical-reagent grade. The standard stock solutions were prepared in double distilled water (ddH_2_O) and stored at 4 °C protected from light with aluminum foil. Standard working solutions of known concentrations were further prepared in volumetric flasks by diluting the stock aqueous solutions with ddH_2_O.

### 2.2. Harvesting and Selection of Plant Feedstocks

The six elected plant feedstocks to manufacture homemade vinegars were apple (*Malus domestica*), raspberry (*Rubus idaeus*), blueberry (*Vaccinium myrtillus*), blackberry (*Rubus fruticosus*), rose hip (*Rosa canina*) and persimmon (*Diospyros kaki*), giving rise to the corresponding fruit vinegars (and designations), respectively: apple vinegar (HMV1); raspberry vinegar (HMV2); blueberry vinegar (HMV3); blackberry vinegar (HMV4); rose hip vinegar (HMV5); persimmon vinegar (HMV6). All the six fruits were obtained in the region of Berovo, Eastern of North Macedonia, during the harvest season (viz., August, September and October of 2017).

Additionally, seven types of commercial fruit vinegars (ComV) were selected and purchased for investigation. The brands (and designations) of the industrially manufactured vinegars were Agrar (ComVA1)—apple vinegar; Vitalia (ComVA2)—apple vinegar; Crystal (ComVA3)—apple vinegar; and Himbeer (ComVR4)—raspberry vinegar, Ceko-fam (ComVA5)—apple vinegar; Samarji (ComVA6)—apple vinegar; Samarji (ComVP7)—plum vinegar. The first four commercial vinegars (ComVR1 to ComVR4) are the most consumed in the domestic market and were acquired in a local supermarket, whereas the last three (ComVR5 to ComVR7) were directly acquired to the producers, who claimed to produce the vinegars according to traditional methodologies.

### 2.3. Sampling Procedures

**Figure 1:**
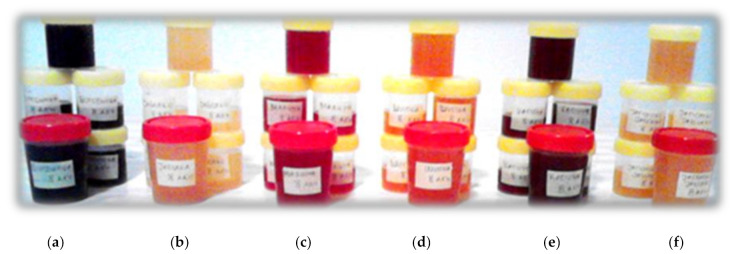
Homemade vinegar (HMV) aliquots at one point of fermentation in closed 50 mL sterile bottles (six samples from double batch per type of fruit): (**a**) blueberry (*Vaccinium myrtillus*); (**b**) apple (*Malus domestica*); (**c**) raspberry (*Rubus idaeus*); (**d**) rose hip (*Rosa canina*); (**e**) blackberry (*Rubus fruticosus*); (**f**) persimmon (*Diospyros kaki*).

Homemade vinegars (HMV1 to HMV6) (Figure 1) were produced in a double batch per type of fruit and aliquots (in duplicate, i.e., two replicates of samples) of 50 mL were taken throughout alcoholic and acetic fermentations at the time periods of 2, 4, 6, 8, 10, 24, 31 and 41 days. After 41 days, the fermentation was stopped by pasteurization at 80 °C for 15–20 min. The vinegars were analyzed after storage with a total lifetime of 60 days (2 months after time zero/day 0). The HMV aliquots were kept in closed 50 mL sterile bottles at −18 °C until further use.

Before analyses, all HMV and Com samples were allowed to warm up to room temperature, centrifuged (Centurion Scientific K3, West Sussex, UK) at 800 rpm for 10 min at room temperature, and further filtered under vacuum through a 90 mm Φ Whatman No. 2 filter paper of 8 µm pore size (Maidstone, UK) and transferred into a 10 mL sterile tube.

### 2.4. Production of Homemade Fruit Vinegars (HMV)

For the production of homemade fruit vinegars, the fresh fruits were selected, washed, dried and cut with removal of the seeds (whenever necessary). About 1400 g of each fruit were then macerated for 1–3 min (Bosch MMB65, Gerlingen, Germany) to expose the chemical constituents as polyphenols, amino acids, oxidative and degradative enzymes, polyphenol oxidase enzyme, lipids, etc. The puree of each fruit was then distributed into 5–6 L glass vessels and 4.8 L of water and 500 g of sucrose were added. The fermentation process was conducted at room temperature, ranging between 21 and 26 °C, for a time period of 41 days in the absence of starter cultures like yeasts and acetic acid bacteria.

Once the acetic fermentation was completed (41 days), the experimental vinegars were filtered, pasteurized (15 min at 80 °C) and transferred, under aseptic conditions, into 100 mL glass airtight containers to reduce the risk of microbial contamination.

### 2.5. Physicochemical Characterization

The content of alcohol in homemade and commercial vinegars was determined according to the AOAC standard method 942.06 (AOAC, 2005) [27], using a Liebig apparatus for distillation.

Acetic acid, as a major organic acid, was determined by titration according to the AOAC standard method 30.071 (AOAC, 1980) [28]. For titratable acidity (TA), 10 mL of each sample was diluted with 20 mL of ddH_2_O and 2–3 drops of phenolphthalein indicator were added afterwards. Acids were titrated with 1 M sodium hydroxide (NaOH). Total acids were expressed as g/L and were calculated using the factor for acetic acid of 0.06.

Soluble dry matter content was determined with a refractometer (Bahco, OIR 32/930050, Sofia, Bulgaria). The pH variation of HMV during the fermentation process and of the final products was measured using a pH meter (SCHOTT, LAB 850, Bath, UK).

Determination of total phenolic content was performed by the Folin–Ciocalteu assay (AOAC SMPR 2015.009: Estimation of total phenolic content using the Folin–Ciocalteu assay, 2015) and expressed as mg/L of gallic acid equivalents [29,30].

Physicochemical analyses of each sample taken in duplicate (biological replicates) were done in triplicate (analytical replicates), thus giving rise to 6 (=2 × 3) observations per sample (see Figure 1) for every fermentation time (*n* = 9 for HMV and *n* = 1 for ComV)—chiefly at 2, 4, 6, 8, 10, 24, 31 and 41 days of fermentation and at 60 days, for HMV, and of the final product for the ComV. As previously described, 6 types of (intermediate and final) HMV and 7 types of final ComV were tested in this research.

### 2.6. Analytical Methods for Determination of the Antioxidant Capacity of Vinegars

In the same way as the physicochemical characterization (see above), analyses for determining the antioxidant capacity of the two replicates of each sample over the time of fermentation (in HMV samples) and in the final product after packing and storage (in HMV and ComV samples) were undertaken in triplicate (analytical replicates).

#### 2.6.1. Cyclic Voltammetry

Cyclic voltammetry was used for the determination of antioxidant capacity of the HMV during the sequential alcoholic and acetic fermentation process. All electrochemical measurements were performed using potentiostat/galvanostat Palm Sens Instrumentation (PalmSens 3, Houten, The Netherlands), controlled by PCTrace version 3.0.6 software (Houten, The Netherlands). The electrochemical cell consisted of a conventional three electrodes set-up. The reference electrode was Ag/AgCl (3 mol/L KCl) (i.e., silver/silver chloride electrode, with 3M of potassium chloride/anion chloride). Platinum wire was used as a counter electrode, while the glassy carbon electrode (GCE, 3 mm-ϕ) was used as the working electrode. The working electrode was cleaned before each measurement by polishing with aluminum oxide (Al_2_O_3_) powder on a polishing cloth for proximately 1 min, followed by rinsing with ddH_2_O and acetone and air-drying. All experiments were performed at room temperature.

#### 2.6.2. Trolox Equivalent Antioxidant Capacity (TEAC) Assay

TEAC assay was performed to determine the antioxidant capacity only in the final HMV and ComV samples. ABTS radical cation (ABTS^•+^) was prepared by reacting 7 mM ABTS stock solution with 2.45 mM ammonium persulfate [(NH_4_)_2_S_2_O_8_] solution. The two solutions were mixed and allowed to stand in the dark at room temperature for a minimum of 12 h in order to produce the ABTS radical cation. The ABTS^•+^ solution was diluted in a ratio of 1:9 (*v*/*v*, 0.7 mM) with ddH_2_O to an absorbance, spectrophotometrically determined, of 0.8 at 735 nm wavelength (Cary 50, Varian, Melbourne, Australia) [31,32].

All vinegar (intermediate and final) samples were filtered through 25 mm Φ disposable syringe filters of 0.45 µm pore size (Chromafil^®^ PET-45/25 Polyester, Macherey-Nagel, Dueren, Germany) into 10 mm micro-cell quartz spectrophotometer cuvettes with PTFE lid (Hellma, Melbourne, Australia). HMV2 and HMV3 samples were diluted 10 times and HMV6 was diluted 15 times with ddH_2_O into screw thread glass tubes to fit within the linearity range of the spectrophotometric method. The decrease of ABTS^•+^ radical absorbance (ABTS decolorization assay) was monitored at 735 nm (visible spectrum) for a time period of 30 min at 37 °C, with an ultraviolet–visible (UV-Vis) spectrophotometer (Cary 50, Varian, Melbourne, Australia), equipped with a multi-cell holder and Cary WinUV Bio Package software. TEAC values were calculated from the Trolox linear calibration curve (square linear correlation coefficient, R^2^ = 0.996) using Trolox standard solutions in the range of 0.5–10 mg Trolox/L sample.

#### 2.6.3. Electrochemical Characterization of 2,2′-azino-bis-3-ethylbenzothiazoline-6-sulfonic acid (ABTS)

Characterization of the electrode reaction of ABTS, i.e., the oxidation of ABTS to a stable ABTS^•+^ radical, was done by means of cyclic voltammetry within the potential range from 0.00 to 0.90 V vs. Ag/AgCl and the scan rate (*v*) interval from 5 to 100 mV/s at glassy carbon electrode (GCE) at ABTS concentration of 0.1 mM. The supporting electrolyte was an aqueous solution containing 1 mM CH_3_COOH and 0.01 M KCl (potassium chloride) as a supporting electrolyte.

**Figure 2:**
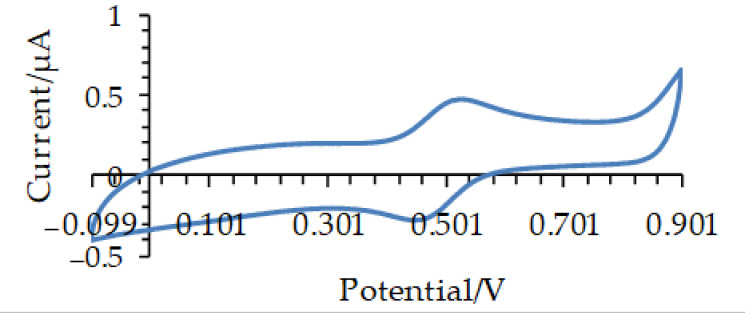
Typical cyclic voltammogram (electric current versus potential) of 0.1 mM ABTS in 1 mM CH_3_COOH and 10 mM KCl at the scan rate of 10 mV/s, with the anodic (Ip,a) and cathodic (Ip,c) peaks in reversible electrochemical processes.

**Figure 3:**
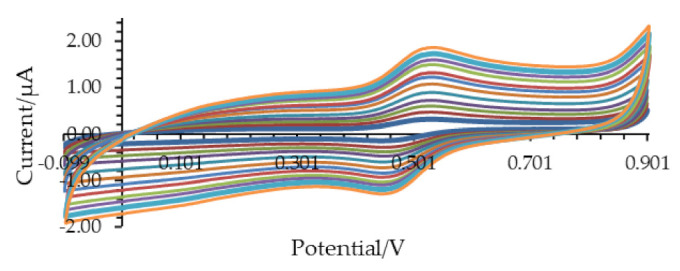
Cyclic voltammograms (electric current versus potential) of ABTS recorded at potential scan rate increasing from 5 to 100 mV/s. The other experimental conditions were the same as in Figure 4.

A typical cyclic voltammogram of 0.1 mM ABTS recorded in a supporting electrolyte containing 1 mM CH3COOH and 10 mM KCl at a scan rate of 10 mV/s is depicted in Figure 2. The shape of the voltammogram is typical for a reversible electrochemical process and encompasses the oxidation of ABTS to a stable ABTS^•+^ radical (anodic peak, Ip,a) and reduction of the radical back to the initial reactant (cathodic peak, Ip,c). Both anodic and cathodic peak currents increased linearly with the square root of the potential scan rate, with a linear correlation coefficient (R^2^) of the linear regression of 0.998 under the above conditions. It implies diffusional mass transfer in the course of the electrochemical experiment as typical for electrode processes of a dissolved redox couple uncomplicated by adsorption phenomena. The ratio between the Ip,a and Ip,c peak currents, measured within the potential scan rate interval from 5 to 100 mV/s, is relatively constant under the above conditions ranging from 0.97 to 1.03, implying electrochemically reversible electrode reaction (Figure 3).

At the same time, the peak-to-peak potential difference between the anodic and cathodic peaks is (60 ± 4) mV, as typical for one-electron reversible electrode reaction. The peak potential difference slightly increases by accelerating the potential scan variation as a consequence of the ohmic drop of the supporting electrolyte.

#### 2.6.4. Determination of Antioxidant Capacity by Cyclic Voltammetry

Antioxidant capacity of all vinegar samples during the fermentation of HMV samples and final products (HMV and ComV samples) was determined by using 5 mL of the sample spiked with 0.1 mM ABTS as redox mediator and 0.01 m KCl as a supporting electrolyte, at the potential scan rate of 10 mV/s. The antioxidant capacity was expressed by estimating the apparent reaction rate constant (*k*_AOX_, expressed in s^−1^) of the homogeneous redox reaction between ABTS^•+^ cation radicals and antioxidants from the vinegars.

**Figure 4:**
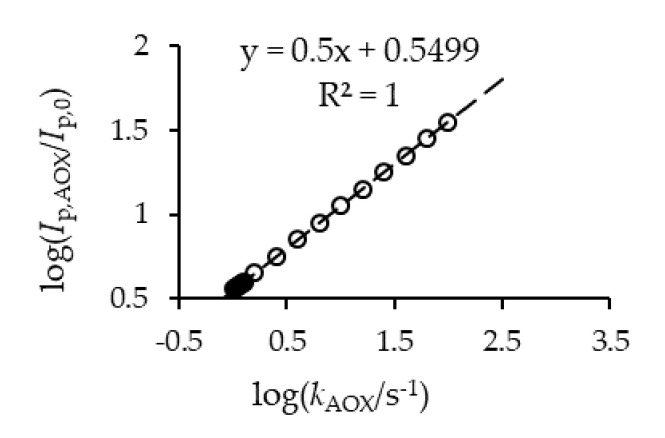
Linear calibration curve and equation constructed by theoretical simulations of the ErC’ reaction mechanism, where log is the base 10 logarithm, Ip,AOX and Ip,0 are anodic peak currents in the presence and the absence of an antioxidant, respectively, k_AOX_ (s^−1^) is the antioxidant capacity constant (or apparent constant) and R^2^ is the square of linear correlation factor.

For calibration purposes, the *E_r_C’* electrode mechanism was theoretically analyzed under conditions of cyclic voltammetry and a theoretical linear calibration curve was constructed as a *log-log* (base 10 logarithm) scale of *Ip*,AOX/*Ip*,0 vs. *k*_AOX_ (Figure 4). Here, *Ip*,AOX and *Ip*,0 are anodic peak currents in the presence and absence of an antioxidant, respectively. The relative, dimensionless nature of the parameter *Ip*,AOX/*Ip*,0 enables direct comparison of the theoretical and experimental data.

### 2.7. Statistical Analysis

For examination of the dependent variables pH value, total acidity (g/L), content of alcohol [ethanol content (%, *v*/*v*)], dry matter (%, *w*/*w*) and antioxidant capacity throughout time in the vinegar samples under scrutiny—HMV samples during fermentation (or intermediate samples) and in the final products of both HMV and ComV—one-way analysis of variance (ANOVA) was used by significance level of all statistical analyses set at α = 0.05. The only studied factor was the fermentation time—more specifically the intermediate samples and final vinegars, i.e., 2, 4, 6, 8, 10, 24, 31 and 41 days of fermentation and at 60 days, for the 6 types of HMV and the final products for the 7 types of ComV. The one-way ANOVA compares the mean values of pH, total acidity, percentage of alcohol, dry matter and antioxidant activity measured through the concentration of ABTS^•+^ by cyclic voltammetry and TEAC assay in order to determine whether there is statistical evidence that the associated population means are significantly different. When the *F*-test led to significant differences (at α = 0.05), the one-way ANOVA using Tukey’s post-hoc tests was used to compare differences in the mean values (in HMV and ComV samples) between groups of the variable time (fermentation time and in storage time after fermentation). An α-value of 0.05 was used as a reference for the *F*- and post-hoc tests.

All the statistical analyses were performed using the SPSS v.16.0 software (IBM Corporation, Chicago, IL, USA). The ANOVA results were classified using superscript letters (a–d), and where different letters mean significant differences among mean values for a *p* < 0.05.

## 3. Results

### 3.1. Physicochemical Characterization of Homemade Fruit Vinegars (HMV) Produced by Double Consecutive Spontaneous Fermentations

**Figure 5:**
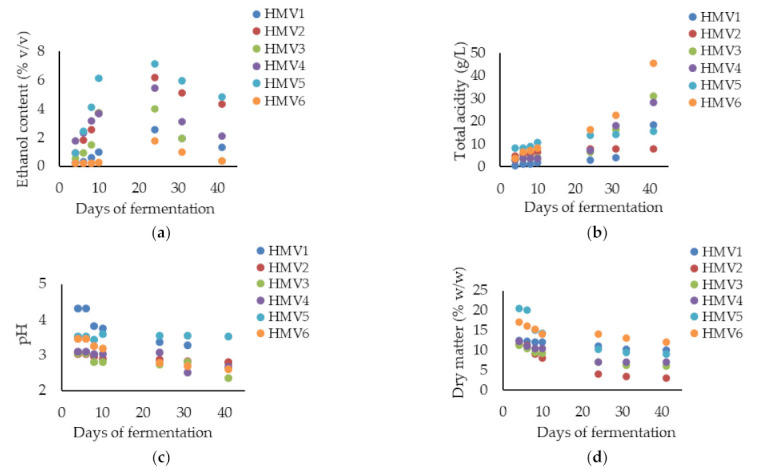
Variation of (**a**) ethanol content (%, *v*/*v*), (**b**) total acidity (g/L), (**c**) pH {−log[H^+^]} and (**d**) dry matter (%, *w*/*w*) in homemade fruit vinegars (HMV) throughout fermentation time (days): apple vinegar (HMV1); raspberry vinegar (HMV2); blueberry vinegar (HMV3); blackberry vinegar (HMV4); rose hip vinegar (HMV5); persimmon vinegar (HMV6). The results were expressed as mean values and standard deviations (mean ± STDV) and calculated from two replicates of each fermentation and three analytical measurements.

During alcoholic fermentation by autochthonous yeasts (Equation (1)), the ethanol level in all HMV samples steadily increased, which happened up to day 24 (Figure 5a) [33]. As a consequence of the loss of metabolic activity by yeasts as ethanol concentration in the fermentative medium increases, the subsequent time period (from 24th to 41st day) was characterized by the consumption (decrease) of ethanol and concomitant production (increase) of acetic acid by the autochthonous AAB present therein (Equation (2))—and which typically became dominant as the spontaneous fermentation proceeds and at the expense of the disappearance of viable yeasts over the fermentation time. Such behavior was observed in all samples with the highest slope for the blueberry (HMV3) and blackberry (HMV4) vinegars (Figure 5a). The levels of ethanol were significantly (*p* < 0.05) found to be the lowest in HMV6 and the highest in HMV5.

As well known, the decrease of ethanol content usually indicates the completion of the alcoholic fermentation by yeasts and the concomitant initiation of acetic fermentation by AAB [34], causing the total acidity to start increasing (Figure 5b) [35]. In the time period from 24th till 41st day, the total acidity rapidly increased, reaching a significant maximum level of 45.6 g/L (*p* = 0.001) for the sample of persimmon vinegar (HMV6). Indeed, the significant (*p* < 0.05) lowest and highest levels of TA were obtained in samples of HMV6 and HMV1, respectively.

During the consecutive spontaneous fermentation processes, pH values of the apple vinegar (HMV1) and blueberry vinegar (HMV3) showed the largest significant decrease (*p* < 0.05), i.e., the highest negative slope of pH values within time (Figure 5c). Furthermore, such a pH decrease was substantially observed as acetic acid fermentation became dominant (approximately from 24th fermentation day onwards). During the alcoholic fermentation time period (approximately from day 1 to 24), the total acid content (Figure 5b) and pH (Figure 5c) tended to stay steady. As seen in Equation (1), the production of carbon dioxide by alcoholic fermentation may further lead to a slight increase of TA and decrease of pH by the formation of hydrogen carbonate (HCO_3_^−^, pK_a2_ = 10.32) or even some carbon acid (or dihydrogen carbonate, H_2_CO_3_, pK_a1_ = 6.37) and possibly also due to some but low AAB activity (with release of acetic acid). Nevertheless, such a decrease is not as significant as the one obtained by the acetic acid (pK_a_ = 4.75) production, since the acidification capacity is much higher [36,37,38,39].

Finally, the content of dry matter in all samples constantly decreased during fermentation. The sharpest decrease was observed for raspberry vinegar (HMV2), i.e., from 11.4 to 3.0% (Figure 5d). In addition, when both consecutive spontaneous fermentations are compared, the decrease of dry matter was more visible during the alcoholic fermentation. In fact, during the acetic acid fermentation, the amounts of dry matter were not significantly different (*p* = 0.07). The significant (*p* < 0.05) lowest and highest levels of dry matter were obtained in samples of HMV2 and HMV6, respectively. The efficiency of alcoholic fermentation was the most noticeable in raspberry vinegar (HMV2) with the lowest percentage of dry matter (3.0%) and the highest amount of alcohol 4.24% [40,41,42,43,44,45].

Overall, the results from Figure 5 revealed a negative correlation (from the 24th day onwards) within the time between total titratable acidity and ethanol and, less substantially, with pH values, whereas a positive correlation was disclosed between ethanol concentration and dry matter.

In the final vinegar products, the pH values were lower than 2.9 but not for the rose hip vinegar (HMV5) (Table 1). According to the Codex Alimentarius Commission (CL 2000/18-EURO, 2000) [46], the pH values in vinegars should be below the tolerance level for several other microorganisms responsible for acidification.

The results from total phenolic content showed that rose hip vinegar had the highest total phenolic content (ca. 20-fold) in comparison to all other vinegars (Table 1). The homemade vinegars from blueberry, blackberry and raspberry had similar values of total phenolic content, while the lowest levels for total phenolic content were determined for persimmon and apple homemade vinegars (Table 1).

### 3.2. Determination of the Antioxidant Capacity of Homemade Fruit (HMV) and Commercial (ComV) Vinegars by Cyclic Voltammetry and TEAC Assay

**Figure 6:**
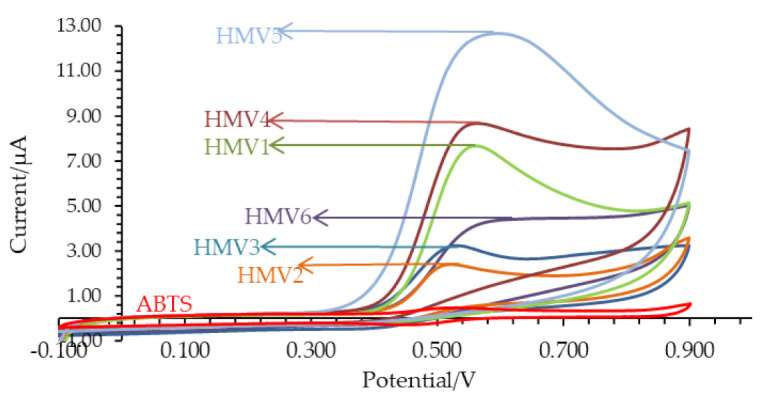
Cyclic voltammograms of homemade vinegars (HMV) after 2 months of fermentation process in relation to the cyclic voltammogram of ABTS (red curve): apple vinegar (HMV1); raspberry vinegar (HMV2); blueberry vinegar (HMV3); blackberry vinegar (HMV4); rose hip vinegar (HMV5); persimmon vinegar (HMV6).

Results presented in Figure 6 revealed high antioxidant capacities of homemade vinegars as final products—thus confirming that spontaneous fermentation induces structural breakdown of fruit cells with the release of fruit antioxidant compounds as well as with the production of the strong antioxidant acetic acid. All studied HMV exhibited well-defined and intensive oxidation voltammetric peaks, particularly (in descending order) for the samples of HMV5, HMV2 and HMV3, whereas lower anodic peak currents are found for HMV4, HMV1 and HMV6.

Electrochemical data was also presented based on the antioxidant capacity constant throughout fermentation time and are summarized in Figure 6. In the initial stage, the 2nd and 4th day of fermentation did not exhibit significant differences between the values of total antioxidant capacity (*p* = 0.071). Afterwards, antioxidant capacity for all samples from the 2nd to 10th day of fermentation exhibited significant variation (*p* = 0.042). At the following period, i.e., from the 17th to 41st day of fermentation, and for the samples HMV2, HMV3 and HMV5, the antioxidant capacity gradually increases (Table 2) until the end of the fermentation period but the antioxidant activity for HMV1, HMV4 and HMV6 had a perceptible variation. The antioxidant capacity of HMV1 and HMV4 was significantly higher (*p* = 0.047 and *p* = 0.032), while the antioxidant capacity of HMV6 did not change significantly at the end of the fermentation process (*p* = 0.075).

**Figure 7:**
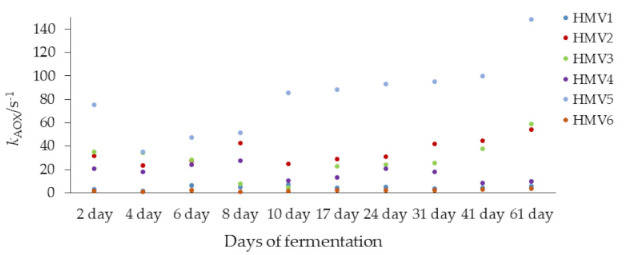
Antioxidant capacity constant (s^−1^) of homemade fruit vinegars (HMV) over time (day), obtained by cyclic voltammetry: apple vinegar (HMV1); raspberry vinegar (HMV2); blueberry vinegar (HMV3); blackberry vinegar (HMV4); rose hip vinegar (HMV5); persimmon vinegar (HMV6).

All samples, but not HMV4, were observed (Figure 7) to entail an increasing trend of antioxidant capacity from the 2nd day to 61st day, as the alcoholic and acetic acid fermentations process. After the fermentation period of 41 days, the vinegars were filtrated and pasteurized at 80 °C for a period of 15–20 min and bottled into plastic bottles (volume 300 mL).

Industrial manufactured ComV (ComVA1, ComVA2, ComVA3 and ComVR4) had significantly lower antioxidant capacity (*p*-values ranging from 0.001 to 0.042) (1.24, 5.8, 0.14 and 3.74 s^−1^, respectively) than ComV samples produced according to the traditional conditions (ComVA5, ComVA6 and ComVP7, with antioxidant capacity of 56.29, 37.32 and 7.57 s^−1^, respectively). Several researchers reported that artificial sweeteners and saccharides were added to the commercial vinegars during the acetic acid fermentation process. This explains why the saccharide content of commercial vinegars was higher than in traditional homemade vinegars [47].

**Figure 8:**
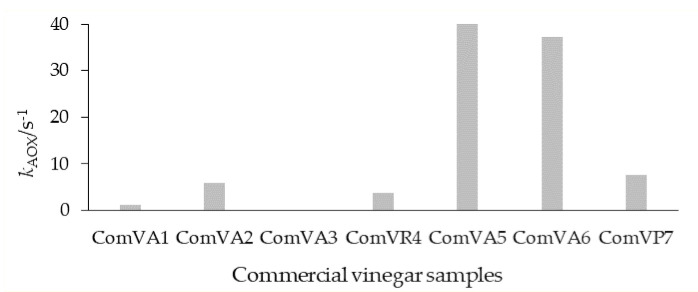
Antioxidant capacity constant (s^−1^) of commercial fruit vinegars (ComV) over time (day), obtained by cyclic voltammetry: Agrar (ComVA1)—apple vinegar; Vitalia (ComVA2)—apple vinegar; Crystal (ComVA3)—apple vinegar; and Himbeer (ComVR4)—raspberry vinegar, Ceko-fam (ComVA5)—apple vinegar; Samarji (ComVA6)—apple vinegar; Samarji (ComVP7)—plum vinegar.

Results presented in Figure 8 indicated that commercial vinegars ComVA5 and ComVA6 presented the highest antioxidant capacity while the antioxidant capacity for commercial vinegar ComVA3 was almost undetectable.

On the other hand, the TEAC values of traditional homemade vinegars, presented in Table 2, were significantly higher in comparison to commercial vinegars. The antioxidant capacities of HMV5, HMV2 and HMV3 were higher than HMV1, HMV4, and HMV6 vinegars. The highest TEAC value was obtained for the sample of rose hip vinegar (HMV5), 10.3 mg/mL TE (Trolox equivalents) (*p* = 0.003), and the lowest value of 0.103 mg/mL TE for the sample of apple vinegar (*p* = 0.049) (HMV1).

The antioxidant capacity of the industrial apple vinegars ComVA1 and ComVA3 were more than 2- and 4-times lower in comparison to homemade apple vinegar (HMV1). The explanation lies in the fact of the higher total phenolic content in HMV in comparison to ComV [48,49,50,51,52,53].

**Figure 9:**
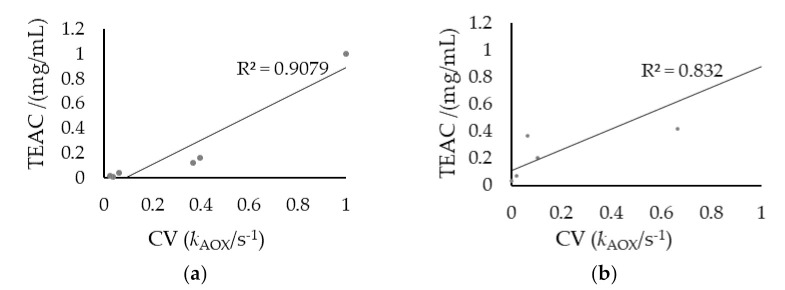
Linear correlation analysis between cyclic voltammetry (CV) and Trolox equivalent antioxidant capacity (TEAC) (mg/mL) assay: (**a**) HMV; (**b**) ComV. CV was expressed as CV (*k*_AOX_), where *k*_AOX_ (s^−1^) is the antioxidant capacity constant (or apparent constant); *R^2^* is the square of linear correlation factor.

Correlation analysis (Figure 9) was applied to the antioxidant capacity estimated by the two methods (cyclic voltammetry and TEAC assay) for the final products of HMV and ComV. A good correlation of antioxidant activity by cyclic voltammetry and TEAC assay was found, with a Pearson coefficient of R^2^ = 0.9079 for HMV (Figure 9a) and with a R^2^ = 0.832 for ComV (Figure 9b). From here, it was possible to conclude that both methodologies are valid for the determination of antioxidant capacity and the use of cyclic voltammetry is a promising methodology due to the features of simplicity, speed and reliability, especially for fermented food. Since yeasts and bacteria (particularly, AAB) are present in the medium during fermentation, cyclic voltammetry is a proper technique for vinegars or other samples designed to entail the growth of those microorganisms [20].

## 4. Discussion

The antioxidant capacity of traditional vinegars produced by fast fermentation processes has been estimated in several studies [54,55,56]. Nevertheless, electrochemical techniques are seldom applied for such purposes—in spite of the fact that they provide a direct insight into the electron-transfer redox-based reaction mechanisms [48,49].

The production of homemade and industrial vinegars is different in the employed procedures. Generally, fruit vinegars entail two major processing steps: one is the production of ethanol from raw materials, and the second is the conversion of the ethanol into acetic acid [12]. Vinegars contain, mainly, acetic acid by weight and small quantities of alcohol, glycerol, esters, sugars and salts. To obtain industrial vinegars made of pure acetic acid, they are subjected to purification by distillation(s). Commercial vinegars are produced either by fast or slow fermentation processes. In the slow or natural processes, vats of cider are allowed to sit open at room temperature [15]. During a period of several months, the fruit juices ferment into alcohol and then oxidize into acetic acid. Slow methods generally are used with traditional vinegars, in which fermentation proceeds slowly over the course of weeks or even months. The longer fermentation period allows the accumulation of a nontoxic slime composed of AAB and soluble cellulose, known as the “mother” (or the starter culture or ferment) of vinegar [57].

Generally speaking, spontaneous fermentation of fruits for vinegar production encompasses initially an alcoholic fermentation for 24 days, where fructose, glucose and sucrose, as most abundant sugars, are broken down into carbon dioxide (CO_2_) and ethanol as main metabolic compounds, as well as other metabolic by-products and volatile compounds in trace amounts. Yeasts’ activity is inhibited by the advent of the increase of ethanol concentration in the fermentation medium by a negative feedback metabolic process. The alcoholic fermentation is followed by the acetic fermentation (by AAB) carried out in the dark from ca. 24 to 41 days, where the ethanol from yeasts is converted to acetic acid and water. The use of dark conditions during vinegar production and manipulation of all antioxidant reactants is important to avoid light oxidation. The alcoholic (Equation (1)) and acetic acid (Equation (2)) exothermic (ΔH < 0) fermentations occur under aerobic conditions and can be simplified by the following chemical equations, respectively:

(1) $$C_{6}H_{12}O_{6}+O_{2}\overset{\Delta H<O}{\to }2C_{2}H_{5}OH+2CO_{2}$$

(2) $$C_{6}H_{5}OH+O_{2}\overset{\Delta H<O}{\to }2CH_{3}COOH+H_{2}O$$

**Figure 10:**
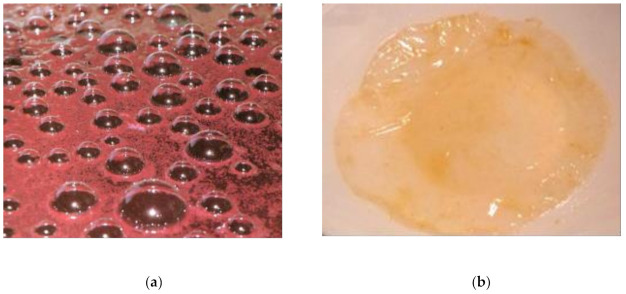
Images of the (**a**) alcoholic fermentation of HMV2 from raspberry and (**b**) fungus on HMV1 from apple.

Although the optimum pH range for acetic acid bacteria growth and multiplication in traditional vinegars is from 5.0 to 6.5, the acetic acid bacteria can survive and stay stable even at much lower pH values as 3 or 4 [58,59,60]. The pH values in the current study were significantly higher than values reported by Kim et al. (2013) [44] and Bakir et al. (2016) [35], due to the dependency on the nature of fruits used (or other matrix whatsoever) for production of vinegars as well as on the specificity of each spontaneous fermentation. Images of the alcoholic fermentation of HMV2 from raspberry and fungus found in apple vinegar on HMV1 from apple are presented in Figure 10a,b, respectively.

According to Jang et al. (2015) [56], during alcoholic fermentation by yeasts in nuruk and koji, sugars were used as fermentation substrates aiming at the biochemical saccharification of starch to alcohol. [56]. Polyphenol compounds are secondary metabolites that have been shown to have high levels of antioxidant activity [19]. Berries, including black raspberry, are well known to contain high levels of quercetin and cyanidin compounds—both of them being well-known as antioxidants [58].

The same analyses for ethanol content, total acids, pH and dry matter during 41 days of fermentation were performed after the time period of two months (60 d)—i.e., the final fruit homemade vinegars. The samples of apple vinegar (HMV1) and persimmon vinegar (HMV6) showed a significantly higher percentage of dry matter in comparison to the other HMV samples because of large amounts of non-fermentable sugars (viz. polysaccharides). Such non-fermentable sugars may be (chemically, enzymatically or microbiologically) converted into fermentable sugars (Table 1) [59,60]. Obtained values for dry matter content are in agreement with Codex Alimentarius Commission (CL 2000/18-EURO, 2000) [46]—which requires minimum values of soluble solids content in fruit vinegars to be not less than 10 g/L per 1% (*v*/*v*) acetic acid. However, the content of total acids in the final vinegars was not in the range of Codex Alimentarius Commission (CL 2000/18-EURO, 2000) [46] recommendation for vinegar production, which requires not less than 40 g/L (calculated as 0.4 g/100 mL of the sample). Yet, more than 1 Vol (%) for ethanol content was detected only for HMV2 and HMV5. According to the Codex Alimentarius Commission (CL 2000/18-EURO, 2000) [46], the content of ethanol in vinegars should be lower than 0.5% (*v*/*v*).

In conclusion, homemade fruit vinegars made by consecutive spontaneous alcohol and acetic acid fermentations need to be optimized, to yield acceptable levels of ethanol and acetic acid based on the referential values from Codex Alimentarius (CL 2000/18-EURO, 2000) [46]. Having in mind that the goal here is to use spontaneous fermentations in the absence of starter cultures, some trials may be performed, for example increasing the time of acetic acid fermentation, changing the fermentation temperatures, manipulating the content of fermentable sugars at the beginning of the alcoholic fermentation or the acidity/pH within fermentation, mixing different raw materials (fruits), among many other possibilities. When the goal is not to use necessarily spontaneous fermentations but instead to use selected autochthonous strains of yeasts and AAB previously isolated from these fruit matrixes, then several trials may be designed and performed, such as selecting the strains based on appropriated phenotype traits (e.g., yields of production and/or tolerance to ethanol and acidity), selecting the most compatible strains in type and/or number of strains and/or ratio of each microbial strain in the designed starter cultures. It can also pass through the independent addition of selected yeast(s) and AAB(s) within time, as well as through the optimization of the amount of inoculum.

The significantly higher pH value of rosehip vinegar (pH = 3.4) can be attributed to the presence of vitamin C and organic acids [61]. The lower values obtained in our vinegars are assumed to be due to the production based on spontaneous fermentation and the low temperature during fermentation. Hence, this research made evident the high dependence between fermentation and composition of the (fermentable) matrixes.

In fact, it is well recognized in the literature the likelihood to positively influence the antioxidant capacity of these vinegars by using different selected strains of *Acetobacter* and yeasts [61]. Such expected influence of the selected strains for the production of vinegars (in addition to the type of the matrixes) may be in the origin of the observed distinct antioxidant capacities in the current research study, especially for HMV1, HMV4 and HMV6 samples [55,62].

Overall, analysis of the antioxidant capacity revealed that traditional homemade vinegars produced by spontaneous fermentations contained successfully higher values of antioxidants in comparison to commercial vinegars [60]. According to Cappozi et al. (2017) [58], spontaneous fermentations at moderate temperature and under limited oxygen access assure a low level of lipid oxidation in vinegars, while maintaining the proper aerobic conditions (aeration) for the alcoholic and acetic fermentations (Equations (1) and (2)). Moreover, due to hydrolytic action of enzymes from the fruit matrixes, the antioxidant capacity of vinegars commonly increases [58].

The total phenolic content (TPC) was determined from water and ethanolic extracts from hips of selected *Rosa* species by Koczka et al. (2018) [54]. According to their findings, water soluble TPC values for analyzed rosehips ranged from 150.8 mg to 299.2 mg GAE/100 g (dry weight). *R. spinosissima* was characterized by the highest phenolic content. Significantly lower values were found both for *R. canina* and *R. rugosa* which showed similar values. Significantly, the lowest TPC level was measured for *R. gallica*. In the water extract of *R. canina*, TPC level proved to be significantly higher in the samples from Koczka (2018) [54]. The results of our study were fully in agreement with the results presented in the work of Kalemba-Drożdż et al. (2020) [50]. Their study showed the highest phenolic content for rose hip extracts. According to the findings of Kalemba-Drożdż et al. (2020) [50], relatively high amounts of vitamin C were found in all rosehip extracts while in raspberry extracts, ascorbic acid was detected in very small amounts. Furthermore, their HPLC analysis for determination of anthocyanins revealed that kuromanin was found in apple peel extracts, except in fermented vinegar. Idaein and cyanidin-3-*O*-glucoside on the other hand, were detected in raspberry extracts while fermented vinegars were characterized by the lowest concentration and the smallest diversity of flavonoids. [51,62]. Other studies showed that raw materials of fruit vinegars (such as apple and blueberry) contain abundant phenolic acids including catechin, syringic acid, gallic acid, chlorogenic acid, epicatechin, caffeic acid, ferulic acid, rutin, protocatechuic acid, and *p*-coumaric acid [62]. In the work of Kelebek et al. (2017) [63], six phenolic acids were identified and quantified in apple vinegar in particular, vanillic, gallic, protocatechuic, chlorogenic acid, caffeic and *p*-coumaric acid. Within the flavonols, phloridzin and phloretin were detected as major compounds in apple vinegars [63].

## 5. Conclusions

This study aimed to compare the production of fruit vinegars by spontaneous fermentation, with the possibility to examine the effect of the fruit matrix on the chemical characteristics of the final vinegars as well as in the antioxidant capacity determined by two different analytical methodologies: cyclic voltammetry and TEAC assay. The results from total phenolic compounds were in strong correlation with the antioxidant capacity of homemade vinegars. The highest amount of total phenolic content (20.2 mg of gallic acid equivalent/mL sample) and the highest value for antioxidant activity determined by the two analytical techniques was detected in rose hip homemade vinegar HMV5. In addition, the antioxidant capacity of commercial vinegars produced, both by traditional and industrial methodologies, was assessed and compared with the homemade fruit vinegars.

In general, the results for apple and raspberry vinegars interestingly indicated that homemade vinegars had more than 10 times higher antioxidant activity in comparison to commercial vinegars. Furthermore, results from our study indicated a large range of antioxidant capacity levels for different commercial vinegars made from apples. The ratio of each variable between apple commercial vinegars ComVA5 and ComVA3 (28.4) indicated the factors quality of raw material as well as the biotechnological processing as the most contributors for the antioxidant capacity in commercial vinegar from the same fruit. Analyzing the cyclic voltammograms, recorded for each vinegar separately, one could conclude that redox mediator ABTS undertakes electrochemical reaction with antioxidants presented in the vinegars. In the presence of a higher concentration of antioxidant compounds, the electrolytic cell recorded an increase in the intensity of the anodic peak currents and a decrease in the intensity of the peak cathode current. The results from the antioxidant capacity of TEAC assay and cyclic voltammetry were almost identical, and positive correlations found between both techniques indicated that the homemade vinegar from rose hip (HMV5) had the highest antioxidant capacity. The lowest amount of phenolic compounds in apple and persimmon vinegars resulted in the lowest antioxidant capacity which led us to the conclusion that the type of the fruit is a limitation factor for high-quality vinegars. Based on this study, the use of cyclic voltammetry is recommended as simple and robust analytical technique for the determination of the antioxidant activity of homemade and commercial vinegars.

## Figures and Tables

**Table 1:** Chemical composition of final homemade fruit vinegars (HMV) after two months (60 d) of fermentation, for ethanol content (% *v*/*v*), total titratable acids (g/L), pH and dry matter (%, *w*/*w*): apple vinegar (HMV1); raspberry vinegar (HMV2); blueberry vinegar (HMV3); blackberry vinegar (HMV4); rose hip vinegar (HMV5); persimmon vinegar (HMV6). The results were expressed as mean values and standard deviations (mean ± STDV) and calculated from one/two replicates of each fermentation and three analytical measurements.

Final HMV	Fruit	Ethanol (%, *v*/*v*)	Total Acid (g/L)	pH	Dry Matter (%, *w*/*w*)	Total Phenolic Content (mg of Gallic Acid/mL)
HMV1	Apple	0.13 ± 0.01 ^d^	20.4 ± 2.1 ^b^	2.7 ± 0.0 ^b^	11.1 ± 1.4 ^a^	0.29± 0.1 ^d^
HMV2	Raspberry	4.24 ± 0.08 ^a^	9.0 ± 0.8 ^c^	2.9 ± 0.1 ^b^	3.0 ± 0.7 ^d^	0.99 ± 0.0 ^b,c^
HMV3	Blueberry	0.47 ± 0.00 ^c^	43.6 ± 4.7 ^a^	2.5 ± 0.0 ^b^	6.2 ± 0.9 ^c^	1.24 ± 0.5 ^b^
HMV4	Blackberry	0.54 ± 0.01 ^c^	42.6 ± 5.1 ^a^	2.3 ± 0.2 ^b^	8.1 ± 1.1 ^b^	1.70 ± 0.8 ^b^
HMV5	Rose hip	3.7 ± 0.07 ^b^	17.4 ± 1.1 ^b^	3.4 ± 0.1 ^a^	9.3 ± 2.3 ^b^	20.2 ± 2.4 ^a^
HMV6	Persimmon	0.34 ± 0.01 ^c^	46.8 ± 6.0 ^a^	2.4 ± 0.0 ^b^	12.1 ± 2.2 ^a^	0.44 ± 0.0 ^d^

Note: Statistical analysis for the final HMV samples. Different superscript letters (^a–d^) mean significant differences among results in the same column at *p* level inferior to 0.05 (*p* < 0.05).

**Table 2:** Trolox equivalent antioxidant capacity (TEAC) (mg/mL) of final homemade fruit vinegars (HMV) (after two months (60 d) of fermentation) and commercial vinegars (ComV). The samples are apple vinegar (HMV1); raspberry vinegar (HMV2); blueberry vinegar (HMV3); blackberry vinegar (HMV4); rose hip vinegar (HMV5); persimmon vinegar (HMV6); Agrar (ComVA1)—apple vinegar; Vitalia (ComVA2)—apple vinegar; Crystal (ComVA3)—apple vinegar; and Himbeer (ComVR4)—raspberry vinegar, Ceko-fam (ComVA5)—apple vinegar; Samarji (ComVA6)—apple vinegar; Samarji (ComVP7)—plum vinegar. The results were expressed as mean values and standard deviations (mean ± STDV) and calculated from two replicates of each fermentation and three analytical measurements.

Figure	Fruit	TEAC (mg/mL)	ComV Sample	Fruit	TEAC (mg/mL)
HMV1	Apple	0.103 ± 0.05 ^c^	ComVA1	Apple	0.049 ± 0.11 ^d^
HMV2	Raspberry	1.286 ± 0.06 ^b^	ComVA2	Apple	0.100 ± 0.15 ^c^
HMV3	Blueberry	1.625 ± 0.07 ^b^	ComVA3	Apple	0.024 ± 0.03 ^d^
HMV4	Blackberry	0.431 ± 0.01 ^c^	ComVR4	Raspberry	0.248 ± 0.02 ^b^
HMV5	Rose hip	10.312 ± 0.05 ^a^	ComVA5	Apple	0.683 ± 0.08 ^a^
HMV6	Persimmon	0.171 ± 0.03 ^c^	ComVA6	Apple	0.286 ± 0.04 ^b^
Acetic acid (100 g/L)	---	0.008 ± 0.0006 ^d^	ComVP7	Plum	0.695 ± 0.06 ^a^

Note: Statistical analysis for the final HMV and ComV samples. Different superscript letters (^a–d^) mean significant differences among results in the same column at *p* level inferior to 0.05 (*p* < 0.05).

## Data Availability

The data presented in this study are available on request from the corresponding author. The data are not publicly available due to privacy restrictions.

## Abbreviations

ABTS	2,2′-azino-bis (3-ethylbenzthiazoline-6-sulphonic acid)
ComV	commercial vinegars
CV	cyclic voltammetry
HMV	homemade fruit vinegars
TEAC	Trolox equivalent antioxidant capacity
